# Pre-treatment nutrition-related indicators and the prognosis of patients with newly diagnosed epithelial ovarian cancer: an ambispective cohort study

**DOI:** 10.3389/fnut.2025.1489934

**Published:** 2025-01-15

**Authors:** Shirui Wang, Jingyu Zhu, Ningjuan Wu, Nannan Wang, Xiaohe Dang, Minyi Zhao, Juan Zhao, Ding Ma, Xiaofeng Yang

**Affiliations:** ^1^Department of Obstetrics and Gynecology, The First Affiliated Hospital of Xi’an Jiaotong University, Xi’an, Shaanxi, China; ^2^Department of Obstetrics and Gynecology, Tongji Hospital, Tongji Medical College, Huazhong University of Science and Technology, Wuhan, Hubei, China

**Keywords:** epithelial ovarian cancer, nutrition-related indicators, first-line chemotherapy response, progression-free survival, overall survival

## Abstract

**Background:**

Few studies have explored the link between nutritional status and prognosis in patients with epithelial ovarian cancer (EOC), and existing findings are controversial. Thus, this study aimed to explore the effects of pre-treatment nutrition-related indicators on the prognosis of patients with newly diagnosed EOC.

**Methods:**

In this ambispective cohort study, 1,020 patients with EOC diagnosed by pathology examination were enrolled and followed-up until December 31, 2023. Univariate and multivariable analyses were conducted on nutrition-related indicators, including body mass index (BMI), albumin (ALB), hemoglobin (Hb), diabetes mellitus (DM), and hyperlipidemia, along with clinicopathological characteristics that might affect patients’ first-line chemotherapy response, progression-free survival (PFS), and overall survival (OS). Survival curves were created using the Kaplan–Meier method. A Cox proportional hazards model was established to obtain hazard ratios (HRs) and 95% confidence intervals (CIs).

**Results:**

The median follow-up duration was 48 months. Compared with patients having normal nutritional indicators, those with hypoalbuminemia had poorer first-line chemotherapy responses. The proportions of those with complete response (CR), partial response (PR), and stable disease or progressive disease (SD/PD) for the ≤30 g/L, 30 < ALB<35 g/L and normal ALB groups were 57.2, 20.6, and 22.2% vs. 62.0, 22.5, and 15.5% vs.79.5, 13.6, and 6.9%. Patients with hypoalbuminemia had shorter median PFS (mPFS): 15 vs. 19 vs. 57 months in the three groups, respectively; and shorter median OS (mOS): 36 vs. 51 vs. 124 months. Patients with hyperlipidemia also exhibited poorer first-line chemotherapy responses; CR, PR, and SD/PD rates for the hyperlipidemia and non-hyperlipidemia groups were 68.9, 19.5, and 11.6% vs. 76.4, 14.7, and 8.9%, respectively, and shorter mPFS (17 vs. 57 months) and mOS (40 vs. 119 months). Patients with anemia had poorer first-line chemotherapy responses; CR, PR, and SD/PD rates for the anemia and non-anemia groups were 68.4, 19.7, and 11.9% vs. 76.2, 14.9, and 8.9%, respectively. All differences were statistically significant (*p* < 0.05). Multivariable analysis identified hyperlipidemia as an independent risk factor for PFS (hazard ratio [HR] = 2.083; 95% CI:1.726–2.514; *p* < 0.001) and OS (HR = 2.158; 95% CI:1.746–2.666; *p* < 0.001), whereas hypoalbuminemia and anemia were not confirmed as independent prognostic factors. This study found no effect of BMI or DM on patient prognosis.

**Conclusion:**

Pre-treatment hypoalbuminemia, hyperlipidemia, and anemia negatively affected the prognosis of patients with newly diagnosed EOC, with hyperlipidemia being an independent risk factor for shorter survival.

## 1 Introduction

Epidemiological data show that in 2022, the global incidence and mortality of ovarian cancer (OC) ranked eighth among female cancers; in China, both ranked ninth (1). The mortality rate of OC ranks first among all gynecological cancers. Epithelial ovarian cancer (EOC) accounts for approximately 90% of all cases of OC. The standard treatment is based on comprehensive staging surgery or cytoreductive surgery, followed by platinum-based chemotherapy. Approximately 70% of patients with EOC relapse within 3 years and often have a poor prognosis owing to platinum resistance or refractoriness (2). The Warburg effect (3), amino acid metabolism (4) and lipid metabolism (5) have been proved to be closely related to tumor growth, metastasis and the regulation of anti-tumor immunity. In patients with cancer, the balance of nutrient metabolism between tumor cells and immune cells in the tumor microenvironment affects the occurrence, development, treatment efficacy, and prognosis of tumors.

Malnutrition is associated with an increased risk of complications and reduced efficacy and tolerance to antitumor therapies (6). Overnutrition, such as a high-fat diet and obesity, can promote metastasis and weaken antitumor immunity in certain types of cancers (7). Many studies have shown that nutritional status is closely related to the prognosis of digestive system cancers (8), breast cancer (9), and lung cancer (10); however, studies related to EOC are few and remain controversial (11–15).

In this ambispective cohort study, we aimed to explore the clinical significance of nutritional status and identify independent nutrition-related risk factors for the prognosis of patients with newly diagnosed EOC to provide a theoretical basis for a better prognosis by improving the nutritional status.

## 2 Materials and methods

### 2.1 Study population and data sources

This study included 1,020 patients newly diagnosed with EOC at the Department of Obstetrics and Gynecology of the First Affiliated Hospital of Xi’an Jiaotong University between January 2010 and December 2021. The inclusion criteria were: pathologically confirmed primary EOC; received comprehensive staging surgery or cytoreductive surgery, followed by regular platinum-based chemotherapy; complete clinicopathological data, pre-treatment laboratory examination data and follow-up data; age ≥ 18 years; and life expectancy≥12 weeks at diagnosis. The exclusion criteria were the presence of other primary malignant tumors, pregnancy, liver and kidney diseases that seriously affect nutrition-related indicators, acute or chronic infectious diseases, hematological diseases, and major trauma. This study was approved by the Ethics Committee of the First Affiliated Hospital of Xi’an Jiaotong University. Given that this study was observational in nature and the patients were enrolled retrospectively and anonymously, the requirement for obtaining consent was waived.

### 2.2 Follow-up and outcome assessment

**Figure 1:**
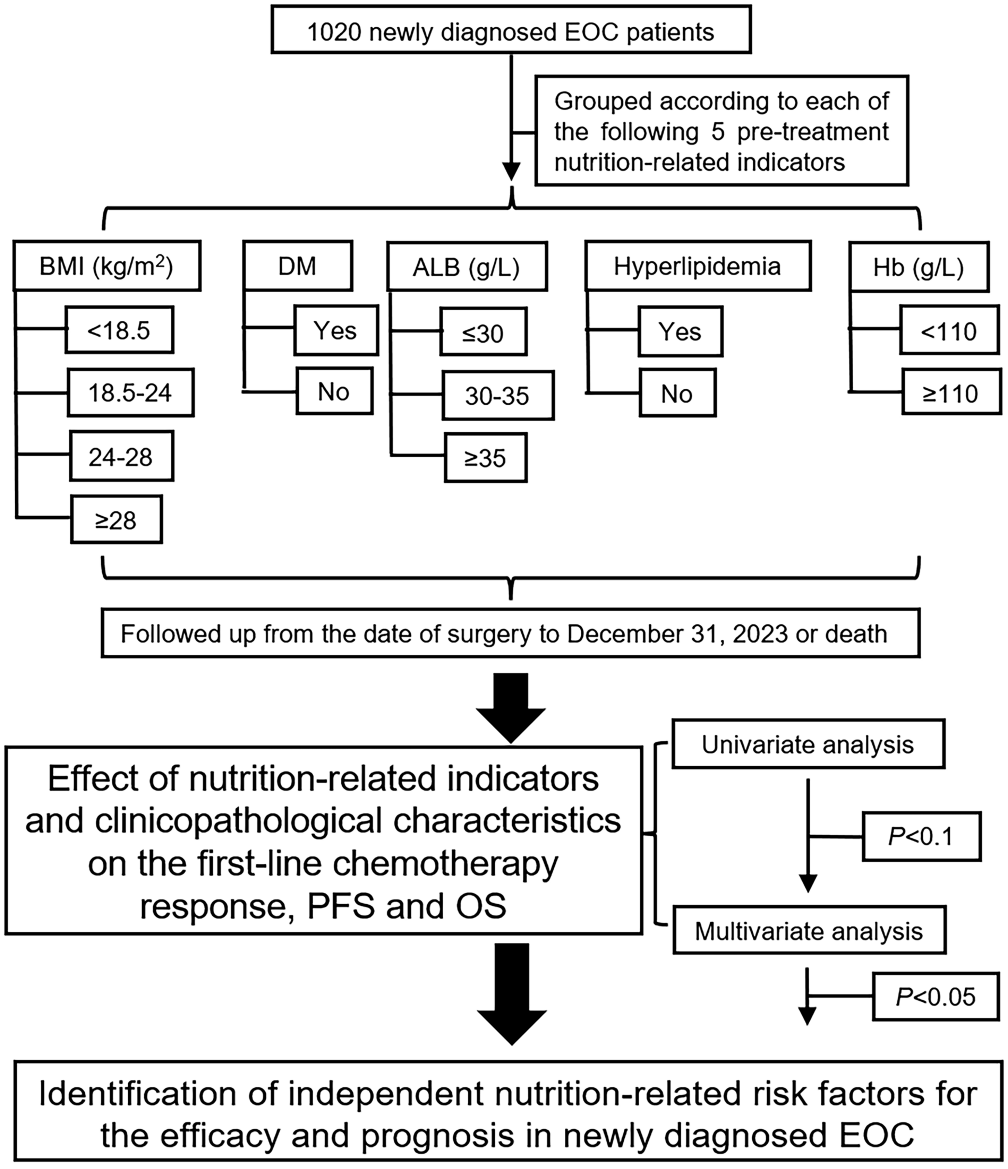
Research flowchart for five nutrition-related indicators to affect the EOC prognosis. EOC, epithelial ovarian cancer; BMI, body mass index; DM, diabetes mellitus; ALB, albumin; Hb, hemoglobin.

In this ambispective cohort study, clinicopathological data and pre-treatment nutrition-related indicators were collected from the electronic medical record system, and patients were divided into groups according to each pre-treatment nutrition-related indicator. All patients were followed up from the date of surgery to December 31, 2023, using the outpatient system. The primary endpoints were overall survival (OS) and progression-free survival (PFS), and the secondary endpoint was first-line chemotherapy response. Figure 1 presents a flowchart of the study.

### 2.3 Definition of related indicators

Five pre-treatment nutrition-related indicators were included in this study. Body mass index (BMI) was calculated as weight/height^2^ (kg/m^2^) and, according to the Chinese BMI standard (16), patients were divided into four groups: BMI < 18.5 kg/m^2^ (underweight), 18.5 ≤ BMI < 24 kg/m^2^ (normal weight), 24 ≤ BMI < 28 kg/m^2^ (overweight), and BMI ≥ 28 kg/m^2^ (obese). Diabetes mellitus (DM) was diagnosed according to the 1999 World Health Organization DM diagnostic criteria (17): patients with typical DM symptoms and random blood glucose≥11.1 mmol/L, or fasting plasma glucose≥7.0 mmol/L, or blood glucose≥11.1 mmol/L at 2-h post glucose load in the oral glucose tolerance test, or glycosylated hemoglobin (HbA1c) ≥ 6.5%. The patients were divided into two groups according to the presence or absence of DM. Patients with EOC complicated by DM included those who had been diagnosed with DM before hospitalization (with a history of DM) and those newly diagnosed with DM after hospitalization (patients whose fasting blood glucose or random blood glucose levels were found to be elevated according to laboratory examinations and who were then diagnosed with DM after consultation with endocrinologists). The normal concentration of serum albumin (ALB) is 35–50 g/L and ALB<35 g/L is diagnosed as hypoalbuminemia; in clinical practice, the indication for ALB administration to patients with cancer is serum ALB≤30 g/L (18); thus, patients were divided into three groups; ALB≤30 g/L, 30 < ALB<35 g/L and ALB≥35 g/L. Patients were divided into anemia and normal groups according to their hemoglobin (Hb) level (19): <110 g/L or > 110 g/L. According to the criteria established by the Joint Committee on the Chinese Guidelines for Lipid Management (20), hyperlipidemia can be diagnosed by the presence of ≥1 of the following four criteria: total cholesterol (TC) > 6.20 mmol/L, triglyceride (TG) > 2.30 mmol/L, low-density lipoprotein (LDL) > 4.10 mmol/L, high-density lipoprotein (HDL) < 1.00 mmol/L. In this study, patients were divided into two groups according to whether they had hyperlipidemia before treatment.

According to the Response Evaluation Criteria in Solid Tumors 1.1 (21) for evaluating the response to first-line chemotherapy in patients with EOC, we divided the patients into three groups: complete response (CR), partial response (PR), and stable disease/progressive disease (SD/PD). OS is the time from the date of surgery to death from any cause, and PFS is the time from the date of surgery to the first time of disease progression or death from any cause.

### 2.4 Statistical analysis

Descriptive statistics of categorical and ordinal categorical variables were expressed as frequency (n) and percentage (%), and descriptive statistics of continuous variables were presented as medians. The Mann–Whitney U test was used for two-group comparisons for univariate analysis of ordinal categorical variables, the Kruskal–Wallis test was used for multiple-group comparisons, with two-by-two comparisons of groups using Bonferroni correction, univariate survival analysis was performed using the Kaplan–Meier method, and the log-rank test was used for comparisons between groups. Factors with *p* < 0.1 in univariate analysis were included in multivariable analysis to screen the independent factors affecting efficacy and prognosis. Ordered logistic regression analysis was used for multivariable analysis of ordinal categorical variables to calculate the odds ratio (OR), and the Cox proportional hazards model was used for multivariable survival analysis to calculate the hazard ratio (HR) and 95% confidence interval (95% CI). Statistical analyses were performed using IBM SPSS Statistics for Windows, version 27.0 (IBM Corp., Armonk, NY, United States). All *p*-values were two-sided, and *p* < 0.05 was considered statistically significant.

## 3 Results

### 3.1 Nutritional status and clinicopathological characteristics

Of the 1,020 patients, 59 (5.8%) were underweight, 569 (55.8%) were of normal weight, 319 (31.3%) were overweight, and 73 (7.1%) were obese. Seventy-five (7.4%) patients had DM. There were 263 (25.8%) patients with hypoalbuminemia, including 63 (6.2%) patients with ALB≤30 g/L and 200 (19.6%) patients with 30 < ALB<35 g/L. Hyperlipidemia was diagnosed in 225 (22.1%) patients. A total of 193 (18.9%) patients had anemia (Table 1).

**Table 1:** Baseline nutritional status and clinicopathological characteristics of 1,020 newly diagnosed EOC patients.

Baseline nutritional status and clinicopathological characteristics		N (%)
Baseline nutritional status		
BMI (kg/m^2^)	<18.5	59 (5.8%)
	18.5–24	569 (55.8%)
	24–28	319 (31.3%)
	≥28	73 (7.1%)
DM	Yes	75 (7.4%)
	No	945 (92.6%)
ALB (g/L)	≤30	63 (6.2%)
	30–35	200 (19.6%)
	≥35	757 (74.2%)
Hyperlipidemia	Yes	225 (22.1%)
	No	795 (77.9%)
Hb (g/L)	<110	193 (18.9%)
	≥110	827 (81.1%)
Clinicopathological characteristics		
Reproductive history	Yes	948 (92.9%)
	No	72 (7.1%)
Menopause	Yes	643 (63.0%)
	No	377 (37.0%)
Family history of cancer	Yes	147 (14.4%)
	No	873 (85.6%)
Age (years)	<60	722 (70.8%)
	≥60	298 (29.2%)
Ascites	Yes	677 (66.4%)
	No	343 (33.6%)
Pathology	HGSOC	673 (66.0%)
	Others	347 (34.0%)
FIGO stage	I	279 (27.4%)
	II	103 (10.1%)
	III	511 (50.1%)
	IV	127 (12.4%)
Residual tumor classification	R0	654 (64.1%)
	R1	275 (27.0%)
	R2	91 (8.9%)
HIPEC	Yes	137 (13.4%)
	No	883 (86.6%)
PARPi	Yes	112 (11.0%)
	No	908 (89.0%)

EOC, epithelial ovarian cancer; BMI, body mass index; DM, diabetes mellitus; ALB, albumin; Hb, hemoglobin; HGSOC, high-grade serous ovarian cancer; FIGO, International Federation of Gynecology and Obstetrics; R0, no residual tumor; R1, microscopic residual tumor; R2, macroscopic residual tumor; HIPEC, hyperthermic intraperitoneal chemotherapy; PARPi, poly ADP-ribose polymerase inhibitors; N, number.

Ascites was found in 677 (66.4%) patients and 673 (66.0%) patients were diagnosed with high-grade serous ovarian cancer (HGSOC). According to the International Federation of Gynecology and Obstetrics (FIGO) staging classification, 279 (27.4%) patients were stage I, 103 (10.1%) patients were stage II, 511 (50.1%) patients were stage III, and 127 (12.4%) patients were stage IV. Surgery achieved no residual tumor (R0) in 654 (64.1%) patients, microscopic residual tumor (R1) in 275 (27.0%), and macroscopic residual tumor (R2) in 91 (8.9%) patients. Among the 1,020 patients with EOC included in this study, 137 (13.4%) received hyperthermic intraperitoneal chemotherapy (HIPEC), and 112 (11.0%) received first-line poly ADP-ribose polymerase inhibitor (PARPi) maintenance therapy (Table 1).

### 3.2 First-line chemotherapy response

#### 3.2.1 Patients with hypoalbuminemia, hyperlipidemia, or anemia demonstrated poorer first-line chemotherapy response compared with those having normal nutritional indicators

After receiving first-line chemotherapy, 762 patients (74.7%) achieved CR, 161 (15.8%) achieved PR, and 97 (9.5%) achieved SD/PD. Univariate analysis showed that patients with hypoalbuminemia had poorer first-line chemotherapy response than those with normal ALB (*p* < 0.001), but there was no significant difference in response between patients with ALB≤30 g/L and 30 < ALB<35 g/L (*p* > 0.05). The proportions of CR, PR, and SD/PD in the ALB≤30 g/L, 30 < ALB<35 g/L and normal ALB groups were 57.2% (*n* = 36), 20.6% (*n* = 13), and 22.2% (*n* = 14) vs. 62.0% (*n* = 124), 22.5% (*n* = 45), and 15.5% (*n* = 31) vs. 79.5% (*n* = 602), 13.6% (*n* = 103), and 6.9% (*n* = 52). Patients with hyperlipidemia had poorer responses than those without hyperlipidemia (*p* = 0.025). The proportions of patients in the two groups were 68.9% (*n* = 155), 19.5% (*n* = 44), and 11.6% (*n* = 26) vs. 76.4% (*n* = 607), 14.7% (*n* = 177), and 8.9% (*n* = 71), respectively. Patients with anemia had poorer responses than those with normal Hb levels (*p* = 0.026). The proportions of patients in the two groups were 68.4% (*n* = 132), 19.7% (*n* = 38), and 11.9% (*n* = 23), vs. 76.2% (*n* = 630), 14.9% (*n* = 123), and 8.9% (*n* = 74), respectively. The effects of BMI and DM on the first-line chemotherapy response in patients were not statistically significant (*p* > 0.05; Table 2).

**Table 2:** Univariate analysis of pre-treatment nutrition-related indicators affecting the first-line chemotherapy response in newly diagnosed EOC patients (*N* = 1,020).

Nutrition-related indicators		N	First-line chemotherapy response			*p*
			CR [n (%)] (*N* = 762)	PR [n (%)] (*N* = 161)	SD + PD [n (%)] (*N* = 97)	
BMI (kg/m^2^)	<18.5	59	42(71.2)	8(13.6)	9(15.2)	0.784
	18.5–24	569	431(75.8)	85(14.9)	53(9.3)	
	24–28	319	236(74.0)	53(16.6)	30(9.4)	
	≥28	73	53(72.6)	15(20.5)	5(6.9)	
DM	Yes	75	55(73.3)	12(16.0)	8(10.7)	0.753
	No	945	707(74.8)	149(15.8)	89(9.4)	
ALB (g/L)	≤30	63	36(57.2)a	13(20.6)a	14(22.2)a	<0.001*
	30–35	200	124(62.0)a,b	45(22.5)a,b	31(15.5)a,b	
	≥35	757	602(79.5)c	103(13.6)c	52(6.9)c	
Hyperlipidemia	Yes	225	155(68.9)	44(19.5)	26(11.6)	0.025*
	No	795	607(76.4)	117(14.7)	71(8.9)	
Hb (g/L)	<110	193	132(68.4)	38(19.7)	23(11.9)	0.026*
	≥110	827	630(76.2)	123(14.9)	74(8.9)	

a, b, and c represent two-by-two comparisons using the Bonferroni correction, the letters of the two groups are different when *p* < 0.05, the letters of the two groups are the same when *p* > 0.05; and *denotes *p* < 0.05. EOC, epithelial ovarian cancer; BMI, body mass index; DM, diabetes mellitus; ALB, albumin; Hb, hemoglobin; CR, complete response; PR, partial response; SD, stable disease; PD, progressive disease; N/n, number.

After undergoing standard first-line chemotherapy, the proportion of patients with FIGO stage I disease achieving CR, PR, and SD/PD were 97.8, 1.8, and 0.4%, respectively. These proportions were 85.4, 6.8, and 7.8%, respectively, in patients with stage II disease, 68.7, 19.6, and 11.7% in patients with stage III disease, and 39.4, 38.6, and 22.0% in patients with stage IV disease. Generally, the higher the stage, the poorer the response to first-line chemotherapy (*p* < 0.001), however, there was no statistically significant difference between stages I and II (*p* > 0.05; Supplementary Table 1).

#### 3.2.2 Multivariable logistic regression analysis did not identify any independent risk factors for the first-line chemotherapy response

**Figure 2:**
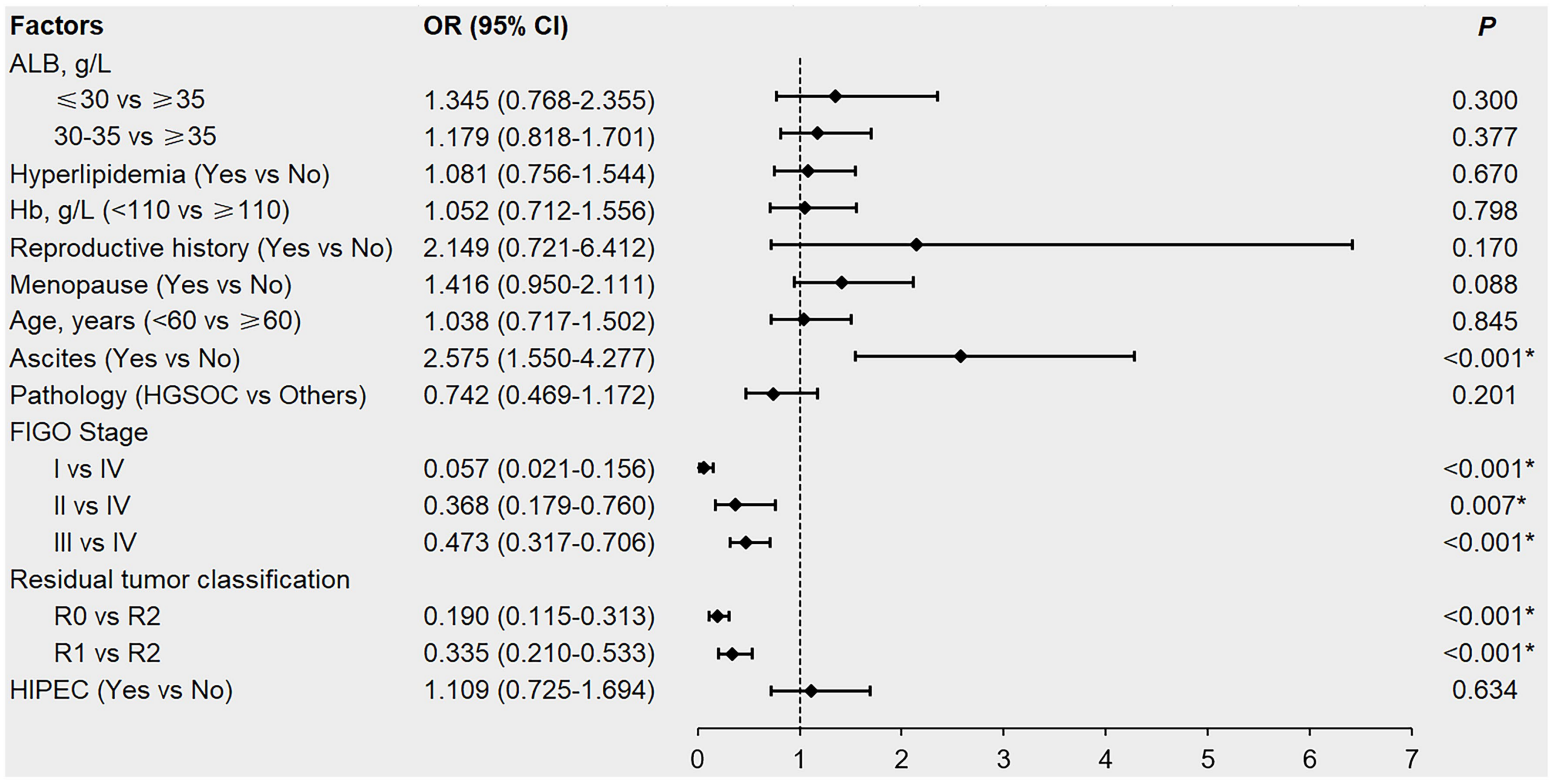
Forest plot of multivariable logistic regression analysis affecting the first-line chemotherapy response in newly diagnosed EOC patients. EOC, epithelial ovarian cancer; ALB, albumin; Hb, hemoglobin; HGSOC, high-grade serous ovarian cancer; FIGO, International Federation of Gynecology and Obstetrics; R0, no residual tumor; R1, microscopic residual tumor; R2, macroscopic residual tumor; HIPEC, hyperthermic intraperitoneal chemotherapy; OR, odds ratio; CI, confidence interval. *denotes *p* < 0.05.

The factors with *p* < 0.1 in the univariate analysis and potentially affecting the first-line chemotherapy response in patients (Table 2; Supplementary Table 1), namely ALB, hyperlipidemia, Hb, reproductive history, menopausal status, age, ascites, pathology, FIGO stage, residual tumor classification, and HIPEC were included in the multivariable logistic regression analysis. Although univariate analysis revealed that pre-treatment hypoalbuminemia, hyperlipidemia, and anemia negatively affected the first-line chemotherapy response in patients with newly diagnosed EOC (*p* < 0.05), multivariable logistic regression analysis failed to confirm these as independent risk factors (*p* > 0.05; Figure 2).

### 3.3 Survival

#### 3.3.1 Patients with hypoalbuminemia or hyperlipidemia had shorter PFS and OS compared with those having normal nutritional indicators

**Figure 3:**
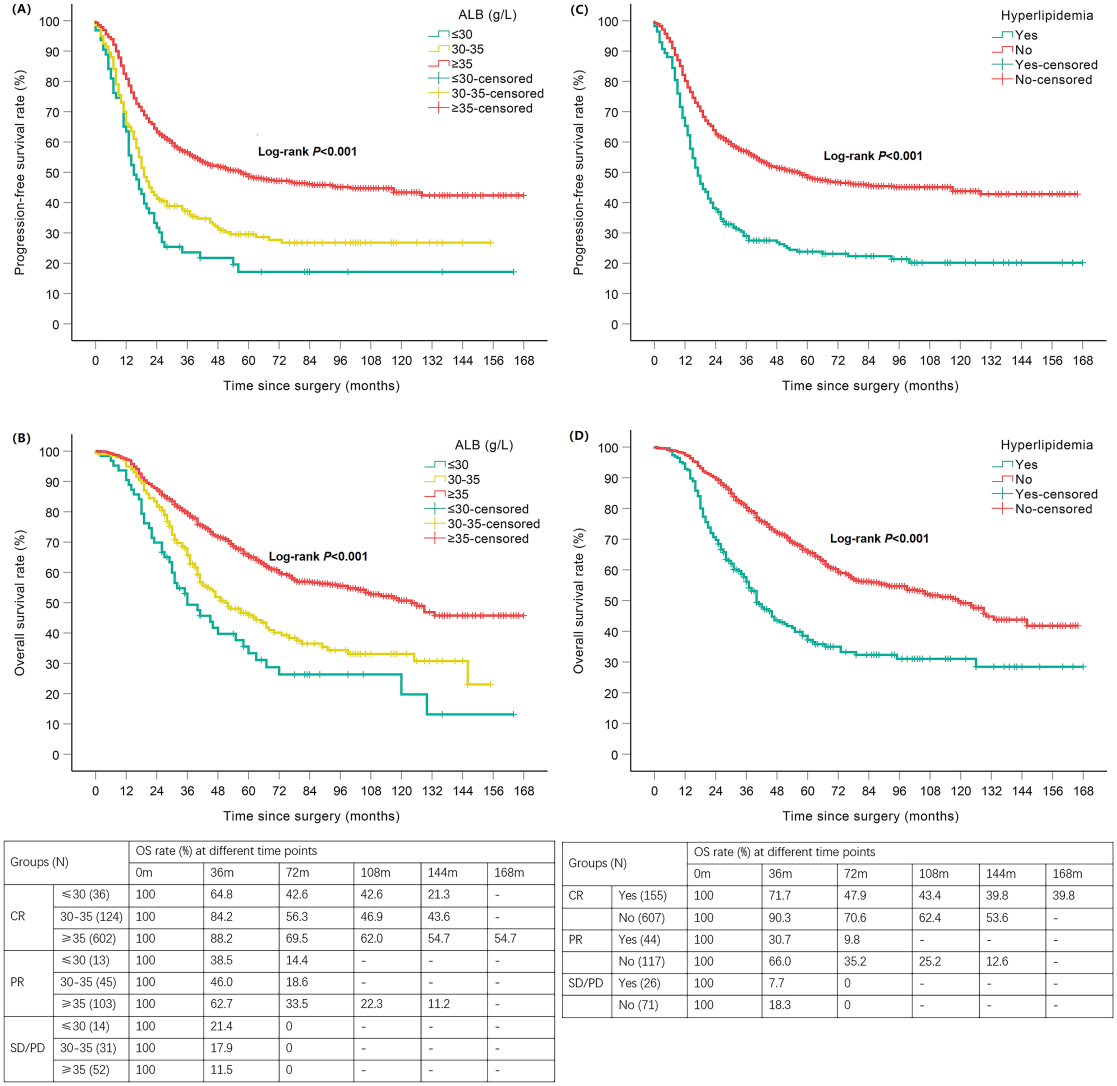
Kaplan–Meier curves of nutrition-related indicators with PFS/OS in newly diagnosed EOC patients. Hypoalbuminemia negatively affected PFS **(A)** and OS **(B)**; Hyperlipidemia negatively affected PFS **(C)** and OS **(D)**. EOC, epithelial ovarian cancer; ALB, albumin; PFS, progression-free survival; OS, overall survival; CR, complete response; PR, partial response; SD, stable disease; PD, progressive disease; N, number; m, months.

The median follow-up for the 1,020 patients was 48 months, during which 580 (56.9%) patients had disease progression and 442 (43.3%) died. Kaplan–Meier survival analysis found that patients with hypoalbuminemia had shorter PFS and OS than those with normal ALB (*p* < 0.001), and patients with ALB≤30 g/L had shorter OS than those with 30 < ALB<35 g/L (*p* = 0.037), but there was no significant difference in the PFS between patients with ALB≤30 g/L and 30 < ALB<35 g/L (*p* > 0.05). The mPFS and mOS of patients in ALB≤30 g/L, 30 < ALB<35 g/L and normal ALB groups were 15 vs. 19 vs. 57 months and 36 vs. 51 vs. 124 months, respectively (Table 3; Figures 3A,B). Patients with hyperlipidemia had shorter PFS and OS than those without hyperlipidemia (*p* < 0.001); the mPFS and mOS of the patients in the two groups were 17 vs. 57 months and 40 vs. 119 months, respectively (Table 3; Figures 3C,D). The effects of BMI, DM, and Hb on PFS and OS were not statistically significant (*p* > 0.05; Table 3).

**Table 3:** Kaplan–Meier survival analysis of pre-treatment nutrition-related indicators affecting the PFS and OS in newly diagnosed EOC patients (*N* = 1,020).

Nutrition-related indicators		mPFS (months)	Log-rank *p*	mOS (months)	Log-rank *p*
BMI (kg/m^2^)	<18.5	23	0.597	58	0.440
	18.5–24	36		79	
	24–28	41		99	
	≥28	42		117	
DM	Yes	37	0.310	72	0.577
	No	38		95	
ALB (g/L)	≤30	15a	<0.001*	36a	<0.001*
	30–35	19a,b		51b	
	≥35	57c		124c	
Hyperlipidemia	Yes	17	<0.001*	40	<0.001*
	No	57		119	
Hb (g/L)	<110	39	0.968	72	0.342
	≥110	37		92	

Log-Rank test was used for comparisons between groups; a, b and c represent two-by-two comparisons between groups, the letters of the two groups are different when *p* < 0.05, the letters of the two groups are the same when *p* > 0.05; and *denotes *p* < 0.05. EOC, epithelial ovarian cancer; mPFS, median progression-free survival; mOS, median overall survival; BMI, body mass index; DM, diabetes mellitus; ALB, albumin; Hb, hemoglobin.

The mPFS of patients with FIGO stages I, II, III, and IV were not reached (NR), NR, 19 months, and 13 months, respectively. Correspondingly, the mOS was 53 months, and 31 months in the NR group. In general, the later the stage, the shorter the PFS and OS (*p* < 0.001). However, there was no statistically significant difference in PFS or OS between patients with stage I and II disease (*p* > 0.05; Supplementary Table 2).

#### 3.3.2 Multivariable cox regression analysis verified hyperlipidemia as an independent risk factor for shorter survival

**Figure 4:**
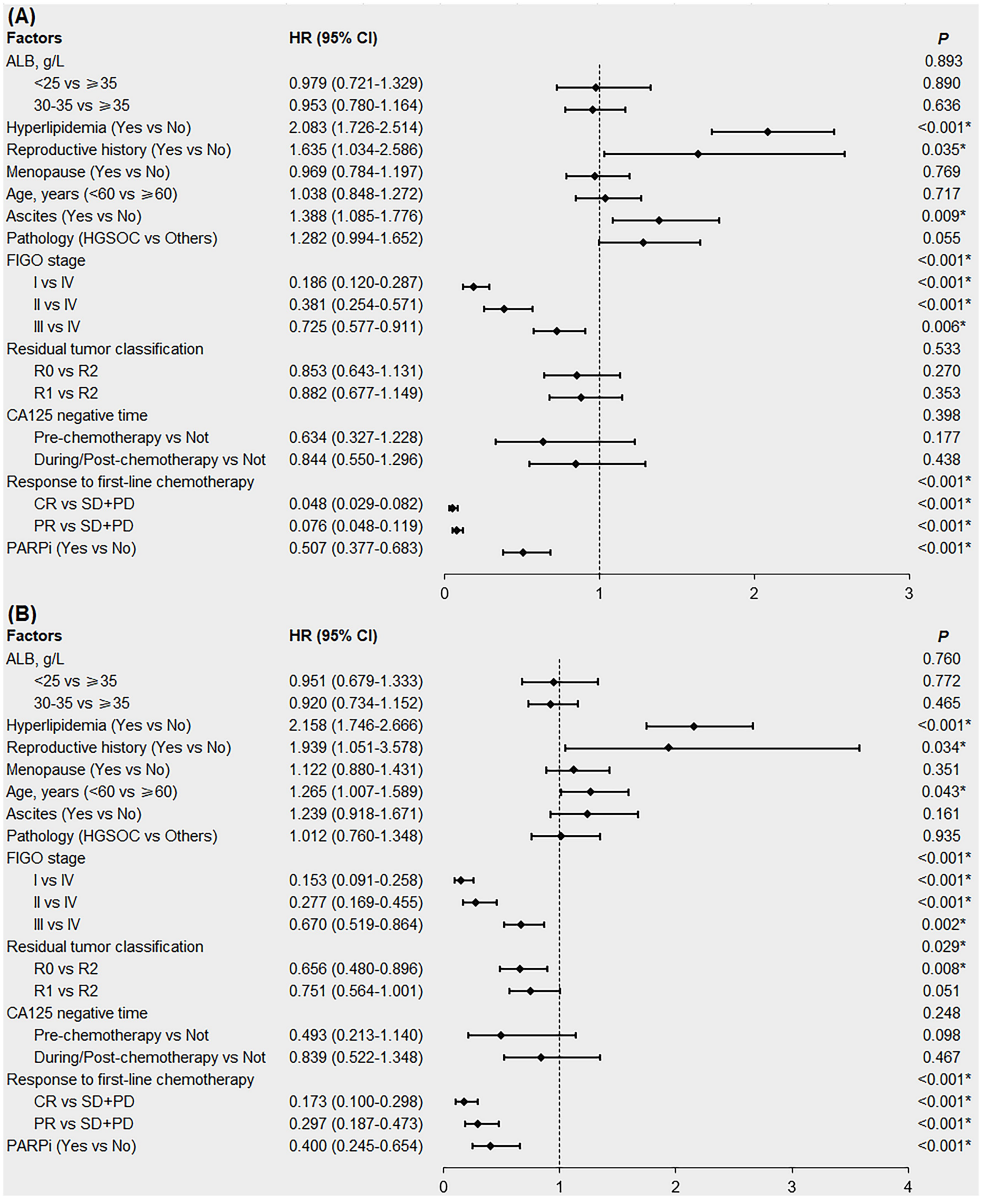
Forest plot of multivariable Cox regression analysis affecting the PFS **(A)** and OS **(B)** in newly diagnosed EOC patients. EOC, epithelial ovarian cancer; PFS, progression-free survival; OS, overall survival; ALB, albumin; HGSOC, high-grade serous ovarian cancer; FIGO, International Federation of Gynecology and Obstetrics; R0, no residual tumor; R1, microscopic residual tumor; R2, macroscopic residual tumor; CA125, cancer antigen 125; CR, complete response; PR, partial response; SD, stable disease; PD, progressive disease; PARPi, poly ADP-ribose polymerase inhibitors; HR, hazard ratio; CI, confidence interval. *denotes *p* < 0.05.

The factors with *p* < 0.1 in the Kaplan–Meier survival analysis and potentially affecting the PFS/OS in patients (Table 3; Supplementary Table 2), namely ALB, hyperlipidemia, reproductive history, menopausal status, age, ascites, pathology, FIGO stage, residual tumor classification, cancer antigen 125 (CA125)-negative time, response to first-line chemotherapy and PARPi were included in the multivariable Cox regression analysis. In patients with newly diagnosed EOC, pre-treatment hyperlipidemia was an independent risk factor for shorter PFS (Figure 4A, HR = 2.083; 95% CI:1.726–2.514; *p* < 0.001) and OS (Figure 4B, HR = 2.158; 95% CI:1.746–2.666; *p* < 0.001). Although univariate analysis showed that pre-treatment hypoalbuminemia negatively affected patient survival (*p* < 0.05), multivariable Cox regression analysis failed to confirm it as an independent risk factor (*p* > 0.05; Figures 4A,B).

We also conducted subgroup analyses according to FIGO stage. The results showed that in patients with early stage EOC (FIGO stages I and II), pre-treatment hyperlipidemia was an independent risk factor for shorter PFS (HR = 1.958; 95% CI:1.179–3.250; *p* = 0.009), but not for OS (*p* = 0.064). In patients with advanced EOC (FIGO stages III and IV), pre-treatment hyperlipidemia was an independent risk factor for shorter PFS (HR = 2.025; 95% CI:1.658–2.473; *p* < 0.001) and OS (HR = 2.118; 95% CI:1.700–2.639; *p* < 0.001; Supplementary Figures 1, 2).

## 4 Discussion

Cancer is a metabolism-related chronic wasting disease. There is growing evidence that the prognosis of patients with cancer is not only related to tumor factors, systemic inflammation, and immune status, but also to nutritional status (22). Malnutrition is found in 32% of patients with cancer, caused by tumor-related anorexia, inflammation, or metabolic changes (6). Several clinical studies have shown that both malnutrition and overnutrition in patients with cancer can affect disease progression and efficacy of anticancer therapy (7, 23). Therefore, evaluation and intervention of the nutritional status of patients with cancer may improve their efficacy and prognosis. There are few studies on the nutritional status and prognosis of patients with EOC; the factors studied are often single indicators or scores, and the results remain controversial. In this study, we used five common pre-treatment nutrition-related indicators, BMI, DM, hyperlipidemia, and ALB and Hb levels, to explore their effects on the prognosis of 1,020 patients with newly diagnosed EOC and identified hyperlipidemia as an independent nutrition-related risk factor for shorter survival.

Globally, there has been a gradual increase in the incidence of being overweight and obese. The Ovarian Cancer Association Consortium (OCAC) combined the data of 12,390 patients with OC from 21 studies and found that a higher BMI was associated with shorter OS and PFS (15). However, a Scottish study involving 1,067 patients with OC found no statistically significant correlation between BMI and survival (11); another study involving patients with EOC reached similar conclusions (24). One study reported that pre-diagnosis BMI ≥ 35 kg/m^2^ was associated with a lower survival rate in patients with stage I-II EOC, but in stage IV patients with BMI ≥ 35 kg/m^2^, the survival rate was higher; this correlation weakened when factors such as ascites and intestinal obstruction were adjusted for (25). However, the relationship between BMI and prognosis in patients with EOC remains controversial. In our study, 319 patients were overweight and 73 patients were obese; however, there was no statistically significant effect of BMI on the prognosis of patients with EOC, which is consistent with most existing studies (11, 24). Obesity is associated with a higher likelihood of comorbid DM, hypertension, and coronary heart disease, while underlying diseases increase the risk of perioperative and chemotherapy complications in patients with EOC, which is not conducive to a good prognosis (26). However, the fat metabolic reserve in obese patients with advanced cancer can help them resist the physiological challenges of surgery and chemotherapy, so those with a high BMI may even have a better prognosis, the “obesity paradox,” this has not been demonstrated in patients with EOC. In addition, although BMI is a commonly used indicator for evaluating the overall nutritional status, body weight is often affected by ascites and cachexia, making BMI unreliable as an appropriate indicator in some patients with EOC. The effect of BMI on the prognosis of patients with EOC, therefore, is uncertain, which may be due to differences in the time of weight measurement, definition of the BMI cutoff, body fat proportion, volume of ascites, and obesity-related underlying diseases. Prospective clinical studies related to overall nutritional status in the future should incorporate BMI, body fat proportion, abdominal circumference, and skinfold thickness simultaneously and choose fasting weight, excluding the effect of ascites, to reduce the error of evaluation and obtain more accurate correlation analysis data.

Glycolytic reprogramming of tumor cells not only provides a large amount of energy but also produces a large number of intermediate metabolites, such as lactic acid, which promote tumor proliferation, invasion, and metastasis (27–29). The Warburg effect also strengthens tumor immune escape and suppresses anti-tumor immunity through local hypoxia, inhibiting the monitoring and lethality of tumor-infiltrating lymphocytes. Inhibiting aerobic glycolysis-related pathways can eliminate tumor growth advantage and immune escape, thereby suppressing the occurrence and development of tumors and promoting anti-tumor immunity (30). A retrospective study has shown that DM is an independent risk factor for the prognosis of patients with EOC (30). However, data from 15 studies conducted by the OCAC showed no correlation between DM and PFS in patients with OC (31). Metformin reduces the production of insulin, insulin-like growth factor, inflammatory cytokines, and vascular endothelial growth factor and has antimitotic, anti-inflammatory, and anti-angiogenic effects (32). In a clinical study, metformin reduced the risk of EOC and prolonged the survival of patients with EOC and DM (33). These findings support the hypothesis that DM influences morbidity and survival in patients with EOC. Of the 1,020 patients included in this study, 75 had DM. No statistically significant correlation was found between DM and the prognosis of patients with EOC. Since metformin use is associated with prolonged survival in EOC patients with DM (33), glycemic control status may affect the prognosis. A history of DM, and levels of blood glucose, HbA1C, and glycated albumin are commonly used to evaluate the nutritional status in relation to carbohydrates. HbA1C reflects glycemic control over the preceding the 3 months, is more stable than other indicators, and is not affected by the external environment, emotion, or current diet; therefore, it is the best indicator for evaluating the status of recent glycemic control. HbA1C has been found to be associated with a risk of recurrence and death in early-stage breast (34) and colon cancer (8). Therefore, the results of this study suggest that, when HbA1C data cannot be used as a reference, the presence of DM does not affect the prognosis of patients with EOC.

In tumor cells, amino acids not only directly participate in the growth and proliferation of tumor cells as raw materials for the synthesis of various proteins or as intermediates for energy metabolism but are also necessary to activate the mammalian target of rapamycin (mTOR) pathway involved in tumor cell proliferation and migration. Additionally, amino acids are indirectly involved in antitumor immunity by regulating immune cell function (35). Amino acid metabolic reprogramming has been shown to be closely related to the development of various cancers (4), so it is particularly important to study the effect of protein nutritional status on patients with cancer. ALB has the highest concentration in human plasma, accounting for more than 60% of the total protein in healthy adults, and not only effectively reflects the nutritional status of patients with EOC but also plays an important role in regulating inflammation, oxidative stress, and innate immunity, and thus affecting the growth of tumor cells (36). Several studies have shown that ALB levels are related to the prognosis of patients with EOC (37–39), however, several questions remain unanswered. First, it is unclear whether hypoalbuminemia is an independent risk factor for the prognosis of patients with EOC. Anorexia in patients with advanced cancer can lead to inadequate amino acid intake and decreased ALB synthesis. High catabolism in patients with cancer leads to increased ALB consumption, and with the progression of the disease, increased capillary permeability leads to the infiltration of ALB into the interstitial space, resulting in the formation of exudates in the serosal cavities. All of the above can cause hypoalbuminemia in patients with cancer; therefore, pre-treatment ALB levels are usually associated with several clinical features. Our study found that compared to the normal ALB group, patients with hypoalbuminemia had a worse prognosis, but this was not an independent predictor of prognosis, which may be because ALB levels were also affected by ascites, body weight, and other nutrition-related indicators. In the Chinese clinical guidelines, the indication for human serum albumin supplementation in patients with cancer is ALB≤30 g/L, while hypoalbuminemia is defined as ALB≤35 g/L (18). Whether ALB in the range 30–35 g/L g/L affects the efficacy and prognosis of patients is clear. Our study revealed that patients with ALB≤30 g/L had shorter OS than those with ALB in the range 30–35 g/L, but the first-line chemotherapy response and PFS were not statistically different between the two groups, which demonstrated that hypoalbuminemia, especially when the ALB level dropped below 30 g/L, might severely affect the long-term survival of patients with EOC. Future clinical trials are needed to evaluate the effect of human serum ALB supplementation on the efficacy and prognosis of patients with EOC, especially those with ALB<30 g/L.

Lipids play a key role in the occurrence and development of tumors, with increased lipid levels supporting the high energy demand of growing tumor cells. Tumor cells reprogram lipid metabolism, mainly by affecting lipid uptake, synthesis, and catabolism. Studies have shown that lipid metabolism-related genes are overexpressed in a variety of cancers to adapt to the high energy demand of tumor cells, and their overexpression has been shown to be associated with poor cancer prognosis. In addition, excessive lipids can affect the function of various immune cells in the tumor microenvironment, thereby inhibiting antitumor immunity (7, 40). The accumulation of unsaturated fatty acids supports the growth and migration of OC cells, leading to worse prognosis. In addition, increased lipogenesis and lipid uptake promote chemotherapy resistance and suppress the immune response required to eliminate tumors (41). Blood lipid levels are commonly used as clinical indicators of lipid nutrition. Although there have been a few clinical studies on the correlation between hyperlipidemia and EOC prognosis, the use of statins after OC diagnosis prolongs survival (14, 42). Our study directly evaluated the effect of pre-treatment hyperlipidemia on the treatment efficacy and prognosis of patients with newly diagnosed EOC. We found that hyperlipidemia was an independent risk factor for poor prognosis, predicting shorter PFS and OS. Patients with hyperlipidemia also had poorer first-line chemotherapy responses than those without hyperlipidemia. However, in the subgroup analysis of patients with early stage EOC, hyperlipidemia was not an independent risk factor for OS. This suggests that pre-treatment hyperlipidemia only affects the short-term survival of patients with early stage EOC but does not affect their long-term survival. A study involving 249 patients with EOC found that the HDL-cholesterol (C)/TC ratio was significantly correlated with chemoresistance and that the HDL-C/LDL-C ratio was an independent protective factor for survival (43). The results regarding hyperlipidemia in our study are consistent with those of that study. Some patients were included in our study retrospectively. Specifically, for patients who had been diagnosed with hyperlipidemia before hospitalization, only their medical history regarding hyperlipidemia was collected during data gathering. Additionally, the lipid profiles of patients without hyperlipidemia were not available. In future prospective studies of patients with EOC, the associations between HDL-C/LDL-C or HDL-C/TC ratios and prognosis should be explored, especially for those with SD/PD.

Anemia is common in patients with cancer. Hb carries oxygen, and anemia is associated with many symptoms such as fatigue, depression, and dyspnea, which seriously affect the quality of life. Hypoxia promotes the metastatic potential and growth of tumor cells, decreases the cellular response to apoptotic signals, and generates therapeutic resistance (44). In addition, anemia itself may induce a feedback mechanism that promotes angiogenesis and leads to a higher proliferation rate of tumor cells (45). Existing studies on Hb levels and prognosis in patients with EOC have small sample sizes, and the results are inconsistent (46). Our study not only analyzed the effect of Hb levels on prognosis but also analyzed the response to first-line chemotherapy. Although the Hb level had no statistically significant effect on PFS or OS, patients with anemia had poorer first-line chemotherapy responses than those with normal Hb levels. Clinical practice should focus on the effects of increased Hb levels during treatment to improve the prognosis of patients with EOC.

The retrospective inclusion of patients, along with the changes in clinical guidelines and testing technologies, has led to certain selection biases and information biases, which in turn have contributed to the limitations of the results in this study. However, our study included patients with newly diagnosed EOC, with a large sample size of 1,020 cases and a median follow-up period of 48 months. Five nutrition-related indicators and three treatment-efficacy or prognostic indicators were analyzed, allowing the effect of nutritional status on patients to be comprehensively considered. Multivariable analysis eliminated the interactions between different types of nutrition-related indicators, as well as between indicators and clinicopathological characteristics, and thus more accurately identified the independent nutrition-related indicator risk factors affecting the treatment efficacy and prognosis of patients with EOC. Our research demonstrated that pre-treatment hypoalbuminemia, hyperlipidemia, and anemia negatively affected the response to first-line chemotherapy in patients with newly diagnosed EOC. Additionally, pre-treatment hypoalbuminemia and hyperlipidemia negatively affected survival, with hyperlipidemia being an independent risk factor for shorter survival. However, BMI and DM did not affect the prognosis of patients with EOC. These five nutritional indicators are easy to assess even in less-developed and resource-limited regions. In future clinical practice, nutritional status intervention during anti-tumor treatment of patients with EOC might improve treatment efficacy and prognosis.

## Data Availability

The original contributions presented in the study are included in the article/Supplementary material, further inquiries can be directed to the corresponding author.
