# Supplementary material for: Pre-treatment nutrition-related indicators and the prognosis of patients with newly diagnosed epithelial ovarian cancer: an ambispective cohort study
Source: Front Nutr. 2025 Jan 15;12:1489934. doi: 10.3389/fnut.2025.1489934 (PMC11774736; doi:10.3389/fnut.2025.1489934)
Supplement: Supplementary file 1 [file Data_Sheet_1.docx]

Supplementary Material

**Supplementary Table 1.** Univariate analysis of clinicopathological characteristics affecting the first-line chemotherapy response in newly diagnosed EOC patients (N=1,020).

| **Clinicopathological characteristics** | | **N** | **First-line chemotherapy response** | | | ***P*** |
| --- | --- | --- | --- | --- | --- | --- |
|  |  |  | **CR**  **[n (%)]**  **(N=762)** | **PR**  **[n (%)]**  **(N=161)** | **SD+PD**  **[n (%)]**  **(N=97)** |  |
| Reproductive history | Yes | 948 | 694(73.2) | 159(16.8) | 95(10.0) | <0.001* |
|  | No | 72 | 68(94.4) | 2(2.8) | 2(2.8) |  |
| Menopause | Yes | 643 | 449(69.8) | 122(19.0) | 72(11.2) | <0.001* |
|  | No | 377 | 313(83.0) | 39(10.4) | 25(6.6) |  |
| Family history of cancer | Yes | 147 | 110(74.8) | 23(15.7) | 14(9.5) | 0.974 |
|  | No | 873 | 652(74.7) | 138(15.8) | 83(9.5) |  |
| Age (years) | <60 | 722 | 564(78.1) | 97(13.4) | 61(8.5) | <0.001* |
|  | ≥60 | 298 | 198(66.4) | 64(21.5) | 36(12.1) |  |
| Ascites | Yes | 677 | 443(65.4) | 147(21.7) | 87(12.9) | <0.001* |
|  | No | 343 | 319(93.0) | 14(4.1) | 10(2.9) |  |
| Pathology | HGSOC | 673 | 454(67.5) | 139(20.6) | 80(11.9) | <0.001* |
|  | Others | 347 | 308(88.8) | 22(6.3) | 17(4.9) |  |
| FIGO stage | I | 279 | 273(97.8)a | 5(1.8)a | 1(0.4)a | <0.001* |
|  | II | 103 | 88(85.4)a | 7(6.8)a | 8(7.8)a |  |
|  | III | 511 | 351(68.7)b | 100(19.6)b | 60(11.7)b |  |
|  | IV | 127 | 50(39.4)c | 49(38.6)c | 28(22.0)c |  |
| Residual tumor  classification | R0 | 654 | 571(87.3)a | 52(8.0)a | 31(4.7)a | <0.001* |
|  | R1 | 275 | 164(59.6)b | 74(26.9)b | 37(13.5)b |  |
|  | R2 | 91 | 27(29.7)c | 35(38.4)c | 29(31.9)c |  |
| HIPEC | Yes | 137 | 90(65.7) | 35(25.5) | 12(8.8) | 0.023* |
|  | No | 883 | 672(76.1) | 126(14.3) | 85(9.6) |  |

a, b, and c represent two-by-two comparisons using the Bonferroni correction, the letters of the two groups are different when *P*<0.05, the letters of the two groups are the same when *P*>0.05; and * denotes *P*<0.05.

Abbreviations: EOC, epithelial ovarian cancer; HGSOC, high-grade serous ovarian cancer; FIGO, International Federation of Gynecology and Obstetrics; R0, no residual tumor; R1, microscopic residual tumor; R2, macroscopic residual tumor; HIPEC, hyperthermic intraperitoneal chemotherapy; CR, complete response; PR, partial response; SD, stable disease; PD, progressive disease; N/n, number.

**Supplementary Table 2.** Kaplan-Meier survival analysis of clinicopathological characteristics affecting the PFS and OS in newly diagnosed EOC patients (N=1,020).

| **Clinicopathological characteristics** | | **mPFS (months)** | **Log-rank *P*** | **mOS (months)** | **Log-rank *P*** |
| --- | --- | --- | --- | --- | --- |
| Reproductive history | Yes | 32 | <0.001* | 77 | <0.001* |
|  | No | NR |  | NR |  |
| Menopause | Yes | 26 | <0.001* | 67 | <0.001* |
|  | No | NR |  | NR |  |
| Family history of cancer | Yes | 27 | 0.368 | 68 | 0.486 |
|  | No | 39 |  | 95 |  |
| Age (years) | <60 | 51 | <0.001* | 120 | <0.001* |
|  | ≥60 | 23 |  | 65 |  |
| Ascites | Yes | 20 | <0.001* | 58 | <0.001* |
|  | No | NR |  | NR |  |
| Pathology | HGSOC | 21 | <0.001* | 58 | <0.001* |
|  | Others | NR |  | NR |  |
| FIGO stage | I | NRa | <0.001* | NRa | <0.001* |
|  | II | NRb |  | NRb |  |
|  | III | 19c |  | 53c |  |
|  | IV | 13d |  | 31d |  |
| Residual tumor  classification | R0 | NRa | <0.001* | NRa | <0.001* |
|  | R1 | 16b |  | 47b |  |
|  | R2 | 11c |  | 29c |  |
| HIPEC | Yes | 28 | 0.203 | 73 | 0.476 |
|  | No | 40 |  | 99 |  |
| CA125 negative time | Pre-chemotherapy | NRa | <0.001* | NRa | <0.001* |
|  | During/Post-chemotherapy | 39b |  | 99b |  |
|  | Not | 5c |  | 21c |  |
| Response to first-line chemotherapy | CR | 117a | <0.001* | 146a | <0.001* |
|  | PR | 17b |  | 40b |  |
|  | SD+PD | 4c |  | 18c |  |
| PARPi | Yes | 48 | 0.062 | NR | 0.004* |
|  | No | 35 |  | 81 |  |

Log-Rank test was used for comparisons between groups; a, b, c and d represent two-by-two comparisons between groups, the letters of the two groups are different when *P*<0.05, the letters of the two groups are the same when *P*>0.05. * denotes *P*<0.05.

Abbreviations: EOC, epithelial ovarian cancer; mPFS, median progression-free survival; mOS, median overall survival; HGSOC, high-grade serous ovarian cancer; FIGO, International Federation of Gynecology and Obstetrics; R0, no residual tumor; R1, microscopic residual tumor; R2, macroscopic residual tumor; HIPEC, hyperthermic intraperitoneal chemotherapy; CR, complete response; PR, partial response; SD, stable disease; PD, progressive disease; PARPi, poly ADP-ribose polymerase inhibitors; NR, not reach.

**Supplementary Table 3.** Kaplan-Meier survival analysis of pre-treatment nutrition-related indicators and clinicopathological characteristics affecting the PFS and OS in early-stage (FIGO stage I and II) EOC patients (N=382).

| **Nutrition-related indicators and**  **clinicopathological characteristics** | | **mPFS (months)** | **Log-rank *P*** | **mOS (months)** | **Log-rank *P*** |
| --- | --- | --- | --- | --- | --- |
| **Nutrition-related indicators** | | | | | |
| BMI (kg/m^2^) | <18.5 | 128 | 0.364 | 133 | 0.135 |
|  | 18.5-24 | NR |  | NR |  |
|  | 24-28 | NR |  | NR |  |
|  | ≥28 | NR |  | NR |  |
| DM | Yes | NR | 0.416 | NR | 0.404 |
|  | No | NR |  | NR |  |
| ALB (g/L) | ≤30 | 56 | 0.082 | 120 | 0.128 |
|  | 30-35 | NR |  | NR |  |
|  | ≥35 | NR |  | NR |  |
| Hyperlipidemia | Yes | NR | 0.001* | NR | 0.023* |
|  | No | NR |  | NR |  |
| Hb (g/L) | <110 | NR | 0.143 | NR | 0.488 |
|  | ≥110 | NR |  | NR |  |
| **Clinicopathological characteristics** | | | | | |
| Reproductive history | Yes | NR | 0.127 | NR | 0.065 |
|  | No | NR |  | NR |  |
| Menopause | Yes | NR | <0.001* | NR | 0.005* |
|  | No | NR |  | NR |  |
| Family history  of cancer | Yes | NR | 0.138 | NR | 0.123 |
|  | No | NR |  | NR |  |
| Age (years) | <60 | NR | 0.123 | NR | 0.134 |
|  | ≥60 | NR |  | NR |  |
| Ascites | Yes | NR | 0.073 | NR | 0.432 |
|  | No | NR |  | NR |  |
| Pathology | HGSOC | NR | <0.001* | NR | 0.002* |
|  | Others | NR |  | NR |  |
| Residual tumor  classification | R0 | NR | 0.418 | NR | 0.664 |
|  | R1 | NR |  | NR |  |
| HIPEC | Yes | NR | 0.482 | NR | 0.402 |
|  | No | NR |  | NR |  |
| CA125 negative time | Pre-chemotherapy | NRa | <0.001* | NRa | <0.001* |
|  | During/Post-chemotherapy | NRb |  | NRb |  |
|  | Not | 3c |  | 24c |  |
| Response to first-line chemotherapy | CR | NRa | <0.001* | NRa | <0.001* |
|  | PR | 117b |  | NRb |  |
|  | SD+PD | 3c |  | 26c |  |
| PARPi | Yes | NR | 0.267 | NR | 0.338 |
|  | No | NR |  | NR |  |

Log-Rank test was used for comparisons between groups; a, b, and c represent two-by-two comparisons between groups, the letters of the two groups are different when *P*<0.05, the letters of the two groups are the same when *P*>0.05. * denotes *P*<0.05.

Abbreviations: EOC, epithelial ovarian cancer; mPFS, median progression-free survival; mOS, median overall survival; HGSOC, high-grade serous ovarian cancer; FIGO, International Federation of Gynecology and Obstetrics; R0, no residual tumor; R1, microscopic residual tumor; HIPEC, hyperthermic intraperitoneal chemotherapy; CR, complete response; PR, partial response; SD, stable disease; PD, progressive disease; PARPi, poly ADP-ribose polymerase inhibitors; NR, not reach.

**Supplementary Table 4.** Kaplan-Meier survival analysis of pre-treatment nutrition-related indicators and clinicopathological characteristics affecting the PFS and OS in advanced (FIGO stage III and IV) EOC patients (N=638).

| **Nutrition-related indicators and**  **clinicopathological characteristics** | | **mPFS (months)** | **Log-rank *P*** | **mOS (months)** | **Log-rank *P*** |
| --- | --- | --- | --- | --- | --- |
| **Nutrition-related indicators** | | | | | |
| BMI (kg/m^2^) | <18.5 | 17 | 0.340 | 49 | 0.517 |
|  | 18.5-24 | 19 |  | 47 |  |
|  | 24-28 | 16 |  | 46 |  |
|  | ≥28 | 19 |  | 49 |  |
| DM | Yes | 18 | 0.651 | 65 | 0.977 |
|  | No | 18 |  | 47 |  |
| ALB (g/L) | ≤30 | 14 | 0.093 | 35 | 0.067 |
|  | 30-35 | 18 |  | 46 |  |
|  | ≥35 | 19 |  | 53 |  |
| Hyperlipidemia | Yes | 14 | <0.001* | 33 | <0.001* |
|  | No | 20 |  | 58 |  |
| Hb (g/L) | <110 | 17 | 0.502 | 47 | 0.903 |
|  | ≥110 | 19 |  | 48 |  |
| **Clinicopathological characteristics** | | | | | |
| Reproductive history | Yes | 18 | 0.080 | 47 | 0.067 |
|  | No | 37 |  | NR |  |
| Menopause | Yes | 18 | 0.233 | 46 | 0.162 |
|  | No | 19 |  | 52 |  |
| Family history  of cancer | Yes | 18 | 0.389 | 47 | 0.706 |
|  | No | 18 |  | 48 |  |
| Age (years) | <60 | 18 | 0.507 | 48 | 0.826 |
|  | ≥60 | 19 |  | 51 |  |
| Ascites | Yes | 17 | <0.001* | 46 | <0.001* |
|  | No | 41 |  | 92 |  |
| Pathology | HGSOC | 18 | 0.122 | 47 | 0.637 |
|  | Others | 19 |  | 52 |  |
| Residual tumor  classification | R0 | 24a | <0.001* | 63a | <0.001* |
|  | R1 | 16b |  | 46b |  |
|  | R2 | 11c |  | 29c |  |
| HIPEC | Yes | 23 | 0.225 | 49 | 0.366 |
|  | No | 18 |  | 47 |  |
| CA125 negative time | Pre-chemotherapy | 38a | <0.001* | 38a | <0.001* |
|  | During/Post-chemotherapy | 21a |  | 58a |  |
|  | Not | 5b |  | 21b |  |
| Response to first-line chemotherapy | CR | 25a | <0.001* | 63a | <0.001* |
|  | PR | 16b |  | 38b |  |
|  | SD+PD | 4c |  | 18c |  |
| PARPi | Yes | 48 | <0.001* | NR | <0.001* |
|  | No | 16 |  | 45 |  |

Log-Rank test was used for comparisons between groups; a, b, and c represent two-by-two comparisons between groups, the letters of the two groups are different when *P*<0.05, the letters of the two groups are the same when *P*>0.05. * denotes *P*<0.05.

Abbreviations: EOC, epithelial ovarian cancer; mPFS, median progression-free survival; mOS, median overall survival; HGSOC, high-grade serous ovarian cancer; FIGO, International Federation of Gynecology and Obstetrics; R0, no residual tumor; R1, microscopic residual tumor; R2, macroscopic residual tumor; HIPEC, hyperthermic intraperitoneal chemotherapy; CR, complete response; PR, partial response; SD, stable disease; PD, progressive disease; PARPi, poly ADP-ribose polymerase inhibitors; NR, not reach.

**
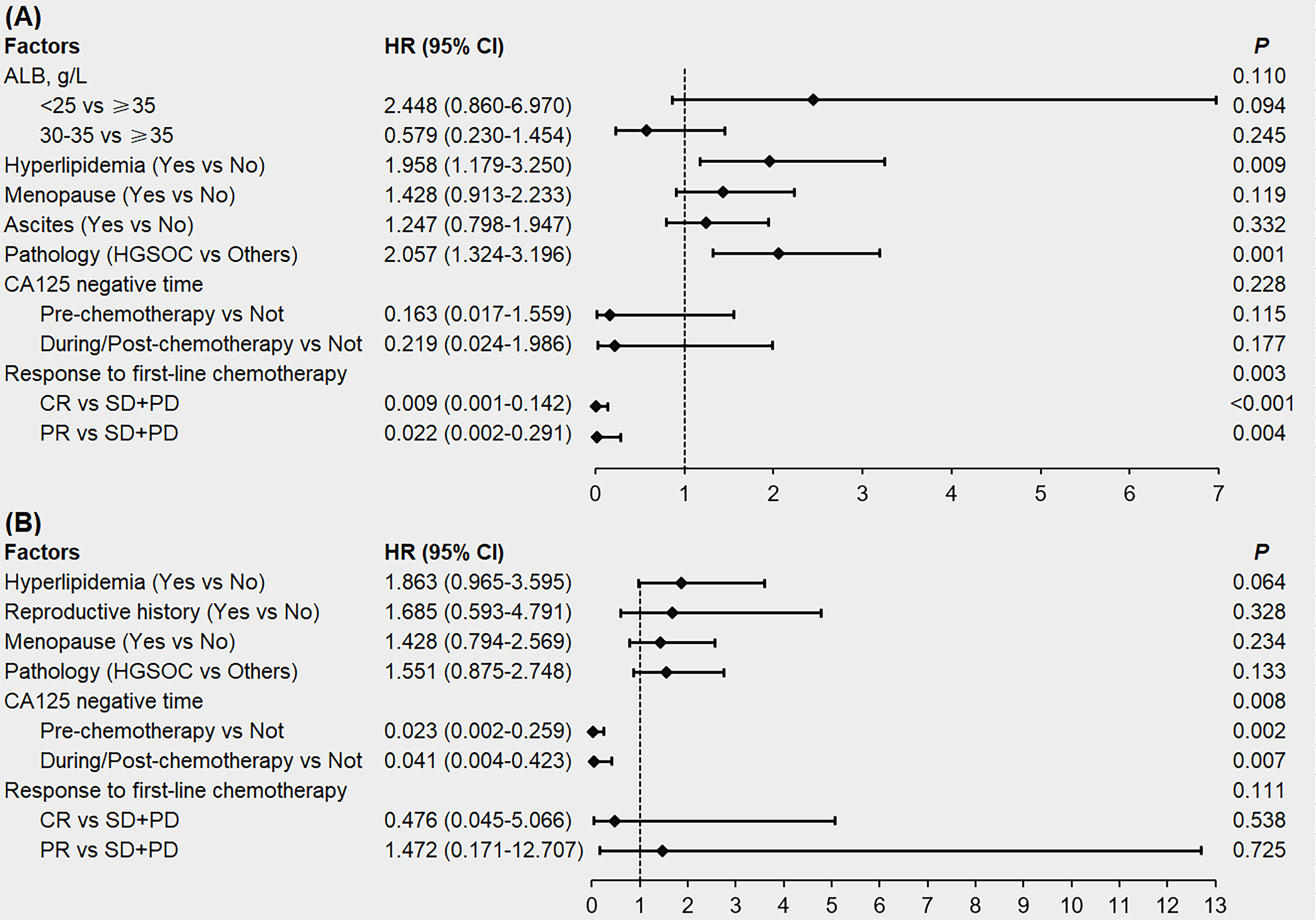
**

**Supplementary Figure 1:** Forest plot of multivariable Cox regression analysis affecting the PFS **(A)** and OS **(B)** early-stage (FIGO stage I and II) EOC patients (N=382).

Abbreviations: EOC, epithelial ovarian cancer; PFS, progression-free survival; OS, overall survival; ALB, albumin; HGSOC, high-grade serous ovarian cancer; FIGO, International Federation of Gynecology and Obstetrics; CA125, cancer antigen 125; CR, complete response; PR, partial response; SD, stable disease; PD, progressive disease; HR, hazard ratio; CI, confidence interval. * denotes *P*<0.05.


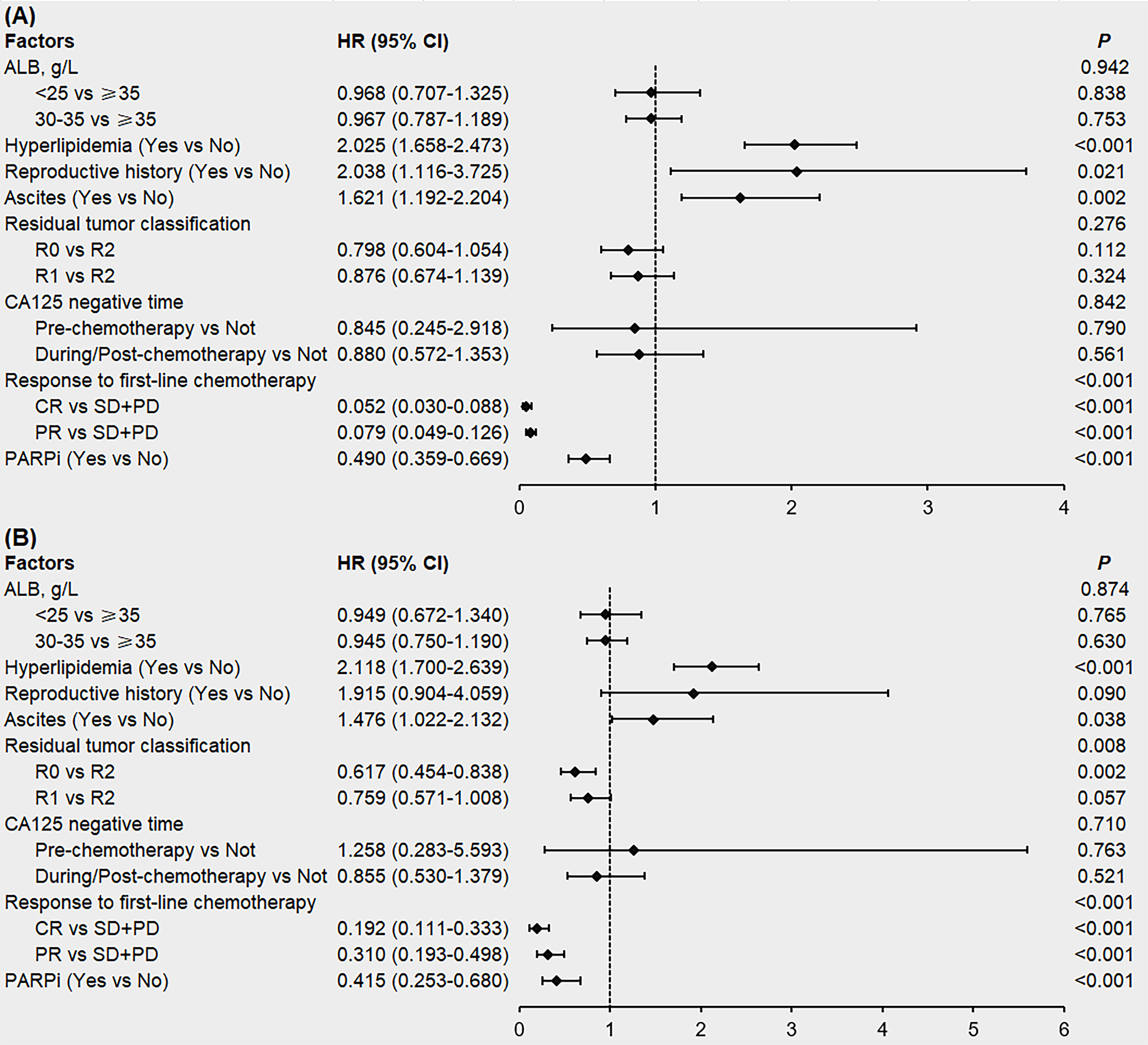


**Supplementary Figure 2:** Forest plot of multivariable Cox regression analysis affecting the PFS **(A)** and OS **(B)** in advanced (FIGO stage III and IV) EOC patients (N=638).

Abbreviations: EOC, epithelial ovarian cancer; PFS, progression-free survival; OS, overall survival; ALB, albumin; FIGO, International Federation of Gynecology and Obstetrics; R0, no residual tumor; R1, microscopic residual tumor; R2, macroscopic residual tumor; CA125, cancer antigen 125; CR, complete response; PR, partial response; SD, stable disease; PD, progressive disease; PARPi, poly ADP-ribose polymerase inhibitors; HR, hazard ratio; CI, confidence interval. * denotes *P*<0.05.
